# Immunotherapy in mastitis: state of knowledge, research gaps and way forward

**DOI:** 10.1080/01652176.2024.2363626

**Published:** 2024-07-07

**Authors:** Afnan Saleem, Sahar Saleem Bhat, Faith A. Omonijo, Nazir A Ganai, Eveline M. Ibeagha-Awemu, Syed Mudasir Ahmad

**Affiliations:** a Division of Animal Biotechnology, SKUAST-K, Srinagar, India; b SKUAST-K, Srinagar, India; c Sherbrooke Research and Development Centre, Agriculture and Agri-Food Canada, Sherbrooke, Canada

**Keywords:** Mastitis, immunotherapy, cytokines, stem cells, exosomes, RNA immunotherapy, epigenetic immunotherapy, bovine

## Abstract

Mastitis is an inflammatory condition that affects dairy cow’s mammary glands. Traditional treatment approaches with antibiotics are increasingly leading to challenging scenarios such as antimicrobial resistance. In order to mitigate the unwanted side effects of antibiotics, alternative strategies such as those that harness the host immune system response, also known as immunotherapy, have been implemented. Immunotherapy approaches to treat bovine mastitis aims to enhance the cow’s immune response against pathogens by promoting pathogen clearance, and facilitating tissue repair. Various studies have demonstrated the potential of immunotherapy for reducing the incidence, duration and severity of mastitis. Nevertheless, majority of reported therapies are lacking in specificity hampering their broad application to treat mastitis. Meanwhile, advancements in mastitis immunotherapy hold great promise for the dairy industry, with potential to provide effective and sustainable alternatives to traditional antibiotic-based approaches. This review synthesizes immunotherapy strategies, their current understanding and potential future perspectives. The future perspectives should focus on the development of precision immunotherapies tailored to address individual pathogens/group of pathogens, development of combination therapies to address antimicrobial resistance, and the integration of nano- and omics technologies. By addressing research gaps, the field of mastitis immunotherapy can make significant strides in the control, treatment and prevention of mastitis, ultimately benefiting both animal and human health/welfare, and environment health.

## 1. Introduction

The mammary gland immune system differs from that of the mucosal organs. The mammary gland has four major roles to play which include: offspring nutrition, passive immunity transfer through immunoglobulin release, immune cells distribution, and self-protection against pathogens. Without jeopardizing the survival of the progeny, the mammary gland has developed to defend itself against infections. Lactating mammary glands exude a nutritious liquid (milk) that collects in the cisterns, collecting ducts, and secretory alveoli before being released during suckling or milking. When colonization by pathogenic microbes takes place, preserving the secretory function remains a major issue since milk as a nutritious liquid supports the rapid growth of microorganisms. Mastitis, the most common and expensive disease in the dairy industry is the outcome, which causes inflammation of the mammary glands. The teat canal forms the first line of defense against invading pathogens by providing a physical barrier and a source of antimicrobial substances. However, once the pathogens breach the teat canal, milk leukocytes form the second line of defense (Nickerson 1985). However, the mammary epithelium is not directly exposed to the outside environment, unlike many other epithelial barriers of the body. The mammary gland lumen is secluded from its environment which in turn is protected by the teat canal, except during nursing or milking. The teat canal integrity is paramount since it delimits the intramammary environment from the mammary gland environment. When the mammary epithelium senses changes in the luminal environment, it functions as an immunologically active barrier, interacting with local and recruited immune cells, to respond to external stimuli (Rainard et al. 2022). Thus, the mammary gland architecture, pathogen type, host factors and other environmental factors play a role in the development of mastitis.

While current management strategies have contributed to decrease mastitis incidence in many countries, they come with unwanted side effects like the potential for the development of antimicrobial resistance (AMR) resulting from frequent antimicrobial use. Other alternative strategies that seek to modulate the immune system to recognize and target specific cells or foreign substances such as pathogens, and collectively known as immunotherapies, do not include the use of antimicrobials and have shown potential as efficient tools for the management of human diseases. However, their application in the management of mastitis is still in the developing phase. The current state of knowledge of available mastitis immunotherapies and their limitations will be presented, as well as the research gaps that should be addressed for the development of more effective immunotherapies.

## 2. Mammary gland inflammation and immunity

### 2.1. Mastitis

Mastitis generally regarded as inflammation of the mammary gland is a complex and costly disease of lactating mammary glands. Mastitis is caused by a plethora of pathogenic microorganisms and its development is determined by the interactions between pathogen (species of microbe), host (breed, genetics, epigenetics, udder structure, age, parity, lactation stage and transition period, etc.), and environmental factors (nutrition, contaminated floor, poor airflow, high stocking density, wet bedding, season, arid and humid climate, etc.) (Contreras and Rodríguez 2011; Dego 2020). Mastitis can range in prognosis, severity and origin depending upon the environment, host-pathogen interactions, and host factors (Schukken et al. 2011; De Vliegher et al. 2012). Penetration of pathogenic bacteria into the mammary gland results in an inflammatory reaction. The inflammatory response is designed to eliminate and neutralize infectious pathogens by preventing colonization and subsequent disease pathology, as well as to promote healing and a return to normal functioning (Sordillo et al. 1997; Aitken et al. 2011). Pathogens causing mastitis can be categorized into contagious and environmental agents depending on their primary reservoir and mode of infection and how they are transmitted (Dego 2020; Martins et al. 2020). The pathogens causing contagious infections thrive on or in the host and spread easily from one infected individual to another, typically through direct or close contact. Among the most common contagious pathogens (*Staphylococcus aureus* (*S. aureus)*, *Mycoplasma bovis (M. bovis*) and *Streptococcus agalactiae, etc.*), *S. aureus* has developed mechanisms to support its persistence and adaptation to host’s physiology for long periods (Zaatout et al. 2020). The environmental pathogens survive and replicate in animal surrounding under various environmental conditions and extended periods, which contributes to their transmission at any point in time. The main environmental pathogens include coliform bacteria (e.g. *Escherichia coli* (*E. coli*)), *Streptococcus spp*. (e.g. *Streptococcus dysgalactiae* etc.) and *coagulase-negative Staphylococcus species* (CNS) (e.g. *S. chromogenes* etc.).

Mastitis can develop as a clinical or subclinical infection depending on the pathogen. Clinical mastitis manifests visible symptoms in an infected cow such as redness and swelling of the udder, udder pain, increased heart beat and change in milk appearance and composition (e.g. watery, bloody, and blood-stained milk as well as clots or flakes in milk) (Blowey and Edmondson 2010). Depending on the degree of inflammation, clinical mastitis can be per-acute, acute or subacute (Fukushima et al. 2020). Subclinical mastitis on the other hand, is the presence of inflammation without any noticeable changes in the milk or udder. If the infection endures beyond a two-month period, it will progress into a chronic state. Occasionally, cows with chronic mastitis exhibit few clinical signs such as fibrosis and fever. Decrease or loss of production and irreversible damage to the affected udder (Martins et al. 2020) are the most pronounced long term effects of chronic infections. An increase in somatic cell count (SCC) is considered an indirect indicator of the presence of infection in the mammary gland.

Vaccination, therapeutic agents (drugs), immunotherapies and breeding for disease resistance have been implemented for the management of mastitis in many countries. These strategies have contributed to the reduction of the incidence of mastitis in dairy herds in Western nations, but they do not efficiently prevent the development of mastitis and its associated production and economic losses. Among them, antimicrobial based drugs (e.g. antibiotics such as penicillin, ampicillin, tetracyclin, gentamycin, etc.) are the most effective and frequently used but concerns about the development of AMR and the presence of AMR genes in animal products and transfer to the food-value-chain necessitate the development of viable alternatives. Immunotherapy on the other hand enhances the ability of the host’s immune system to recognize, target and eliminate pathogens and foreign substances or cells but its application in the management of mastitis is still in the development phase. Thus, enhancing the animal’s natural defense mechanism as well as breeding for mastitis resistance are practical ways of dealing with the disease in dairy herds worldwide. To effectively develop an immune therapy however, deep insights of the host immune system response to pathogens is warranted.

### 2.2. Mammary gland immunity

The combination and coordination of both specific and nonspecific protective aspects, such as the gland’s structural characteristics as well as cellular and humoral defense components, are necessary for the mammary gland immunity (Sordillo et al. 1997). When stressed or during the peri-parturient stage (drying-off and parturition), the immunological defense of the mammary gland is often weakened, which makes them more susceptible to mastitis (Pyörälä 2003; Martin et al. 2018). Mastitis as a complex disease requires the interaction of numerous factors including environment and management practices to either enhance exposure of host to pathogenic agents, lower host natural resistance, or facilitate pathogen entry into the mammary gland environment.

The innate and adaptive (or acquired) immune systems make up the two main components of the mammary immune system. After a pathogenic challenge, the innate immune response is triggered within seconds to minutes (Table 1). Adaptive immunity, however, can take several days to completely activate and generate a more targeted response. Both of these subsystems must be highly interactive and synchronized to offer the best defense against mastitis-causing pathogens. Pathogen recognition receptors (PRR) and the capacity to develop a proinflammatory response are two essential elements of the host innate immune response that are intended to detect and subsequently eliminate infections (Sordillo and Streicher 2002; Paape et al. 2003).

**Table 1.** Mammary gland innate immune defense mechanisms.

Factor	Functions
Teat end	Contraction of sphincter muscles prevents bacterial entry Bacteriostatic property of keratin provides physical obstruction
Pattern recognition receptors (PRRs)	PRRs recognize the bacterial Pathogen-associated molecular pattern molecules (PAMPs) and ultimately, lead to activation of inflammatory responses
Epithelial cells	Pathogen recognition through pattern recognition receptors (PRRs)
Complement system	Bacteriolytic property; phagocytic property
Lactoferrin	Iron sequestration which is needed for bacterial growth
Endothelial cells	Regulated blood flow to the affected areas Leukocyte activation and migration regulation
Oxylipids	Microvasculature regulation Orchestrates proinflammatory and pro-resolving responses
Cytokines	Immune regulation for innate immune responses
Neutrophils	Phagocytosis. Intracellular killing of bacteria through the production of defensins, reactive oxygen species (ROS) and antibacterial enzymes
Dendritic cells	Cytokine production Phagocytosis
Macrophages	Phagocytosis Intracellular bacterial killing Immunoregulatory cytokines and oxylipid production Cellular debris production
Natural killer cells (NK)	Eliminate infected host cells Antibacterial proteins secretion upon activation

Once bacteria enter the teat canal, mastitis is initiated and characterized by varied inflammatory responses as directed by the casual pathogen. The epithelial cells and the resident leukocytes trigger an inflammatory response in order to get rid of the invading pathogen (Aitken et al. 2011). At the site of infection, chemo attractants are released to hasten the recruitment of Polymorphonuclear neutrophils (PMN) and, consequently the increase in milk SCC (Sordillo and Nickerson 1988; Paape et al. 2002). This significant rise in milk SCC after infection is mostly related to an influx of leukocytes from blood into the mammary gland, particularly neutrophils, macrophages, erythrocytes, and lymphocytes (Pyörälä 2003; Souza et al. 2012). Neutrophils phagocytose and kill invading organisms through the action of superoxide ions, hydrogen peroxide, hypochlorite, hydrolytic enzymes, and other soluble defense factors such as defensins and lactoferrin (Riollet et al. 2001; Akers and Nickerson 2011). Milk contains a diverse spectrum of components associated with the innate immune response, including cellular defense components (e.g. leukocytes) and humoral defense components (e.g. complement systems), immune-modulators (anti- and pro-inflammatory cytokines), and chemokines like IL-8 and RANTES, transferrin, lactoferrin, lysozyme, and components of the lactoperoxidase/myeloperoxidase systems that are important in attracting leucocytes from the bloodstream, which significantly aids the host’s capacity to combat the invasive pathogens. The harmful pathogens are then subsequently eliminated by a complex network of cellular and molecular mechanisms (Paape et al. 2003; Ballou 2012).

#### 2.2.1. Cellular defenses of the mammary gland

**Figure 1.**
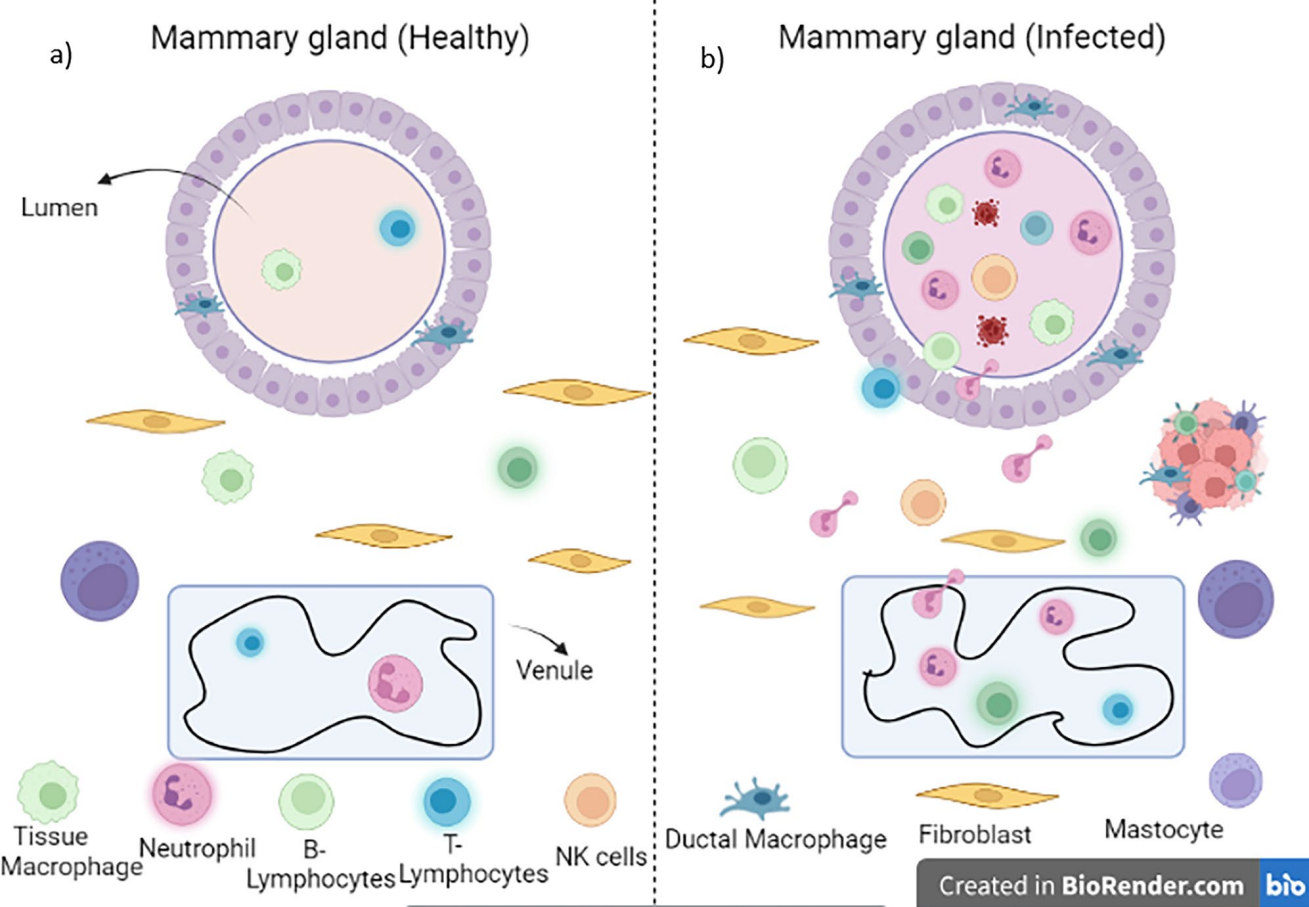
Immune system (steady state) of healthy and infected mammary gland. (a) Mammary gland (healthy) is relatively poor in leucocytes. Mammary epithelium is a bilayer epithelium with cisterns and large ducts. It has ductal macrophages and a few lymphocytes (CD8^+^ T cells). CD4^+^ T cells are dispersed along with stromal macrophages and a few lymphocytes in the Sub-epithelial stroma; (b) in an infected mammary gland, reactive leukocytosis occurs inflammatory cells are recruited by an infected mammary gland which includes lymphocytes, neutrophilic granulocytes, and monocytes. During a chronic infection, aggregation of lymphocytes occurs leading to an inducible tertiary lymphoid formation.

The milk SCC mostly composed of epithelial cells and leukocytes (mainly macrophages, neutrophils, and lymphocytes) are increased during mastitis (Figure 1).

##### 2.2.1.1. Neutrophils

**Figure 2.**
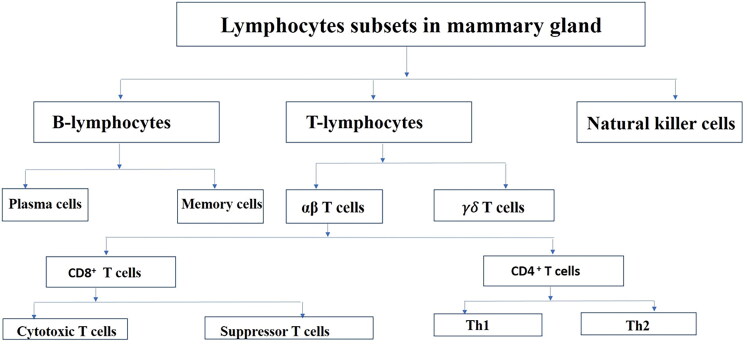
Subsets of mammary gland lymphocytes (adapted from Outteridge and Lee 1981; Riollet et al. 2002; Taylor et al. 1994).

Polymorphonuclear neutrophils (PMNs) are the body’s second line of defense against mastitis. In healthy mammary glands, PMNs constitute 5–25% of cells, increasing to 90% during mastitis (Bradley and Green 2005). They eliminate pathogens *via* oxygen-dependent and independent mechanisms, modulate vascular permeability, and release inflammatory mediators, coordinating innate and adaptive immune responses (Bank and Ansorge 2001; Linde et al. 2008). Thus, PMNs secrete a variety of immune-related factors such as cytokines, chemokines, bactericidal proteins (e.g. defensins, cathelicidins, lactoferrins, and proteases) and peptides in the mammary gland. Once the PMNs perform their tasks, PMNs undergo apoptosis and are cleared by macrophages to prevent further tissue damage (Riollet et al. 2001). **Macrophages:** Macrophages support both innate and acquired immunological responses, specifically through antigen processing and presentation to lymphocytes in conjunction with major histocompatibility complex (MHC) class II antigens (Politis et al. 1992; Fitzpatrick et al. 1992). Macrophages can perform non-specific functions such as ingestion, phagocytosis and destruction of invading bacteria (e.g. *S. aureus*) (Denis et al. 2006), and ingestion of cellular debris and accumulated components of milk in involuting mammary gland (Outteridge and Lee 1981). **Lymphocytes:** T-lymphocytes, B-lymphocytes, and natural killer (NK) cells are three discrete lymphocyte subgroups that function in the mammary gland (Figure 2). Mid-lactation cells have cytotoxic potential and express interferon-𝛾 (IFN-𝛾), while postpartum, CD8+ cells have no cytotoxic activity and express interleukin 4 (IL-4) (Shafer-Weaver and Sordillo 1997). The collaboration between B-cells, MHC class II molecules, and T-helper cells ensures that the immune system can effectively recognize, process, and respond to a wide variety of pathogens (Sordillo and Streicher 2002). Compared to T-lymphocytes, the proportion of B-lymphocytes is rather consistent regardless of infection or lactation stage (Bradley and Green 2005). Natural killer cells are the lymphoid progenitors which generate B- and T-lymphocytes (Roitt et al. 1994). Natural killer cells kill both Gram-positive and Gram-negative bacteria, lyse target cells through antibody-dependent cell-mediated cytotoxicity, exocytosis of granules, cytolytic factor release, and receptor-mediated antigen recognition (Shafer-Weaver et al. 1996).

#### 2.2.2. Soluble components of the mammary gland defense system

The most specific humoral factor in adaptive immune defense are the immunoglobulins (Igs) (Stelwagen et al. 2009) which prevent the adhesion of microbes to tissues, agglutinate bacteria, inhibit bacterial metabolism accelerate opsonization and bacterial phagocytosis (Korhonen et al. 2000). Complement components constitutes an important bridge between the innate and adaptive immune systems, and is involved in initiation and inflammation control, bacterial opsonization, phagocytes recruitment (C3a and C5a), ingestion/killing of microorganisms by phagocytes (C3 and C4) (Barrio et al. 2003; Griesbeck-Zilch et al. 2008). Cytokines regulate inflammation locally and systemically, bridging innate and adaptive immunity (Redpath et al. 2001). Chemokines attract neutrophils and mononuclear cells to infection sites *via* receptors like CXCL1, CXCL2, CXCL3, and CXCL8 ^38^. Host defense peptides (HDPs) are innate immune effector molecules which are predominantly synthesized in PMNs and epithelial cells (Moussaoui et al. 2004; Cormican et al. 2008).

#### 2.2.3. Sensing bacterial microbe-associated molecular patterns by the mammary epithelium

Pathogen threat recognition is a prerequisite for the immune response initiation as well as the mobilization of defenses. This is accomplished by the identification of pattern recognition receptors (PRRs) which are conserved microbial molecules referred to as microbe-associated molecular patterns (MAMPs) (Kumar et al. 2011). The main PRRs families are toll-like receptors (TLRs), retinoic acid inducible gene-1 (RIG-1) and nucleotide-binding oligomerization domain (NOD)-like receptors (NLRs) (are cytosol sensors such as NOD1 and NOD2), each of which recognizes specific bacterial motifs. Microbe-associated molecular patterns associated with common mastitis-related Gram-positive bacteria and Gram-negative bacteria include lipopolysaccharide (LPS) and lipoteichoic acid (LTA), respectively. Bacteria are directly exposed to mammary epithelial cells (MECs) and thus, sensing of MAMPs by MECs of the mammary gland is extensively studied. Cultured MECs are said to have the molecular machinery needed to detect and respond to infections. Overexpression of inflammatory cytokines and chemokines, or TLR4 downstream signaling molecules was observed upon challenge of primary bovine MECs (pbMECs) with *E. coli* or *S. aureus* (Lahouassa et al. 2007; Mumtaz et al. 2022; Taban et al. 2023) or challenge of MEC with LPS (Ibeagha-Awemu et al. 2008). Lipopolysaccharide from Gram-negative bacteria and LTA from Gram-positive bacteria, respectively, are recognized by TLR4 and TLR2 *via* the lipid anchor (Ray et al. 2013). Within the mammary gland, several epithelial sensors are activated simultaneously by the bacterial invader MAMPs which leads to a synergistic response. Microbe-associated molecular patterns have a synergistic effect on pbMECs, causing a robust transcriptome response that includes increased expression of inflammation-associated genes as well as a downregulation of casein gene expression (Wu et al. 2020). The degree of inflammation is determined by the PRRs activation by different ligands and within different cellular compartments, i.e. whether cell surface, cytosolic, or vesicular PRRs are implicated in infections (Barton 2008).

#### 2.2.4. Inflammasome activation by bacteria

**Figure 3.**
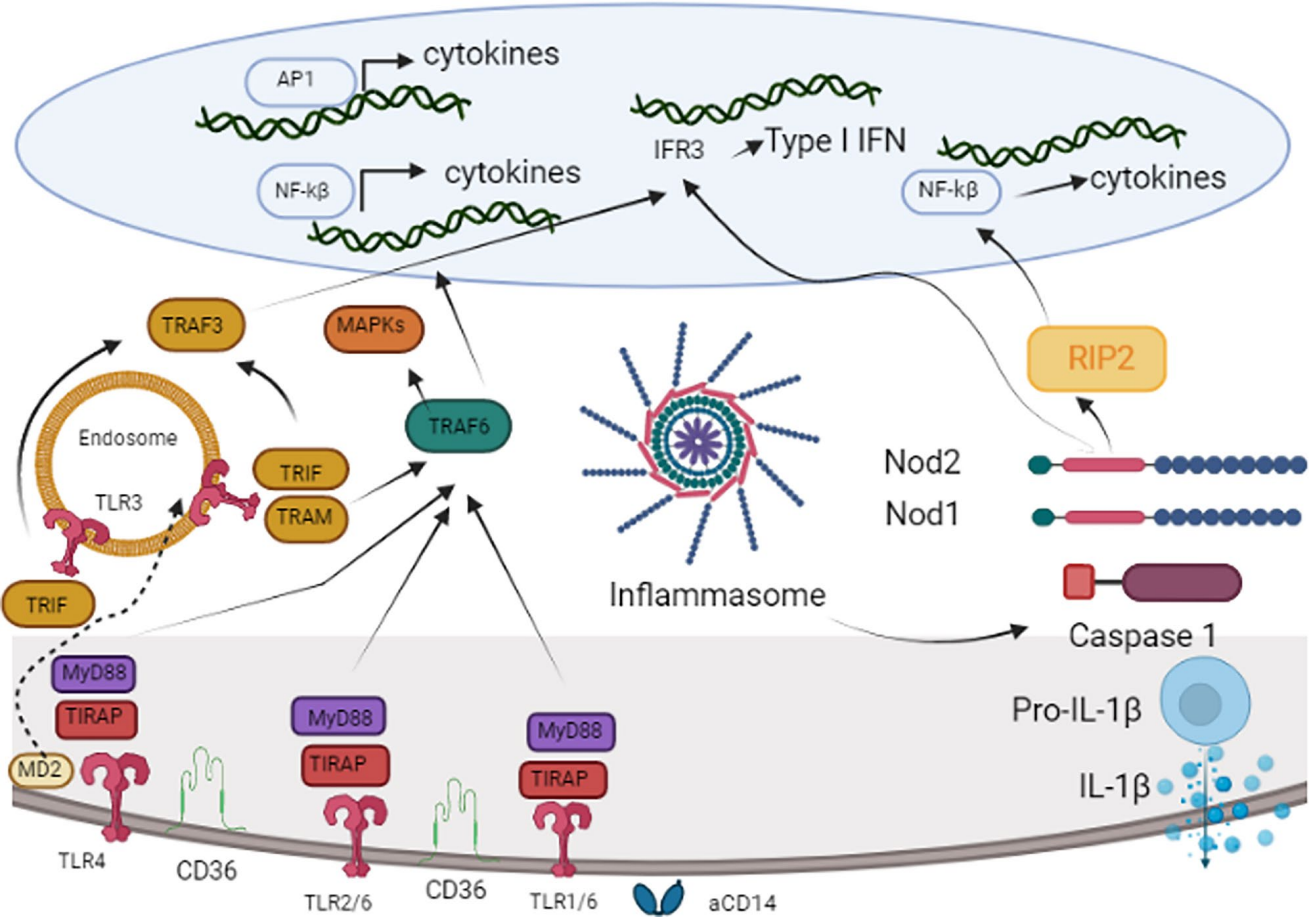
Invading bacteria sensing by (MECs) in the mammary gland. (TLR1), TLR2, TLR4 and TLR6 are exposed at the cell’s apical side. TLRs use accessory molecules that interact with bacterial lipoproteins and LPS, CD36 and MD2. Milk contains sCD14, which is otherwise absent in MECs. As a result, smooth LPS can be internalized by TLR4 upon ligation, and from there, TRAF3 can be activated by attracting TRAM-TRIF adaptors. Additionally, TRAF3 can be activated by TLR3 from endosomes. The TRIF-dependent signaling pathway stimulates IRF3 translocation, which results in the production of type 1 IFNs and the expression of IFN-inducible genes. MAP kinase Cascade activates the transcription factor AP-1 which further leads to cytokine gene activation. TLRs rely on MyD88 adaptor molecule except TLR3 which initiates TRAF6. This allows NF-kβ translocation into the nucleus which activates cytokine gene transcription. MECs responds to bacterial cell wall peptidoglycan degradation products *via* NOD1 and NOD2 cytosolic sensors. When these sensors oligomerize, the adaptor protein RIP2 is recruited, activating the NF-kβ pathway. NLRP3 inflammasomes recruits caspase 1 which contributes to formation of mature pro-inflammatory cytokine IL-1β. MECs: mammary epithelial cells; TLRs: toll like receptors; LPS: lipopolysaccharide; CD36: cluster of differentiation 36; MD2: myeloid differentiation factor 2; sCD14: soluble cluster of differentiation 14; IRF3: IFN-regulatory factor 3; IFN: Interferon gamma; IL-1β: Interleukin-1 beta; NOD: nucleotide-binding oligomerization domain; MYD88: myeloid differentiation primary-response protein 88; RIP2: Receptor-interacting-serine/threonine-protein kinase 2; TRIF: Toll/IL-1 receptor (TIR) domain-containing adaptor protein inducing IFNβ; TRAM: TRIF-related adaptor molecule; TRAF3: TNF receptor associated factor 3; AP-1: Activator protein 1; NF-kβ: Nuclear factor-kappa β; NLRP3: NLR family pyrin domain containing 3.

Inflammasome activation is critical in defending against bacterial infections by developing effective innate immune responses. They regulate bacterial burden as well as the magnitude and nature of adaptive immune responses (Patel et al. 2017; Evavold and Kagan 2018). Inflammasomes are multimeric intracellular signaling protein complexes that include an innate immune sensor, the adaptor protein apoptosis-associated speck-like protein including CARD (ASC), and the inflammatory caspases-1 (CASP1) and/or −11 (CASP11). Caspases, when activated, cleave family cytokines in the cytosol. Pro-form ILs (such as IL-1 and IL-18) are cleaved into their bioactive forms. Downstream of inflammasome activation, active CASP1 or CASP11 cleaves N-terminal of gasdermin D (GSDMD) and generates an N-terminal cleavage product known as GSDMD-NT, translocate it to the cell membrane forming pores by oligomerizing and binding to phospholipids in the cell membranes and leading to cell death, in a process known as pyroptosis (Rühl et al. 2018; Downs et al. 2020; Keestra-Gounder and Nagao 2023) as summarized in Figure 3. Dysregulated inflammasome activation can cause tissue damage in host due to an exaggerated innate immune response. Gram-positive pathogens employ a variety of virulence strategies to elude detection by inflammasomes in host cells and prevent the induction of pyroptosis (Keestra-Gounder and Nagao 2023).

#### 2.2.5. Enhancing the T cell responsiveness of the mammary gland

Adaptive immune response in the mammary gland can be elicited by local immunization as it is provided with antigen-presenting cells which are linked to the systemic mechanisms. Luminal exposure to antigens in a non-lactating gland will generate an optimal immune response eliciting neutrophilic inflammation and enhanced local defense. This results from the activation of resident memory lymphocytes in the mammary gland producing IFN-γ and/or IL-17 ^53^. However, induction of resident memory T cells is the best way to enhance the epithelial barrier and neutrophilic inflammation efficiency and thus, controlling the bacterial infection before it establishes into a persistent infection. The advantage of T cell immunity is that the T cell peptide epitopes are conserved and often shared between bacterial strains which provide a wider protection than antibodies (Chen et al. 2011; Kumar et al. 2013). Induction of mammary gland immunity with antigen-experienced T cells has been exploited in various mastitis vaccination strategies (Rainard et al. 2022; Rainard et al. 2022).

## 3. Mastitis immunotherapy and other strategies

**Figure 4.**
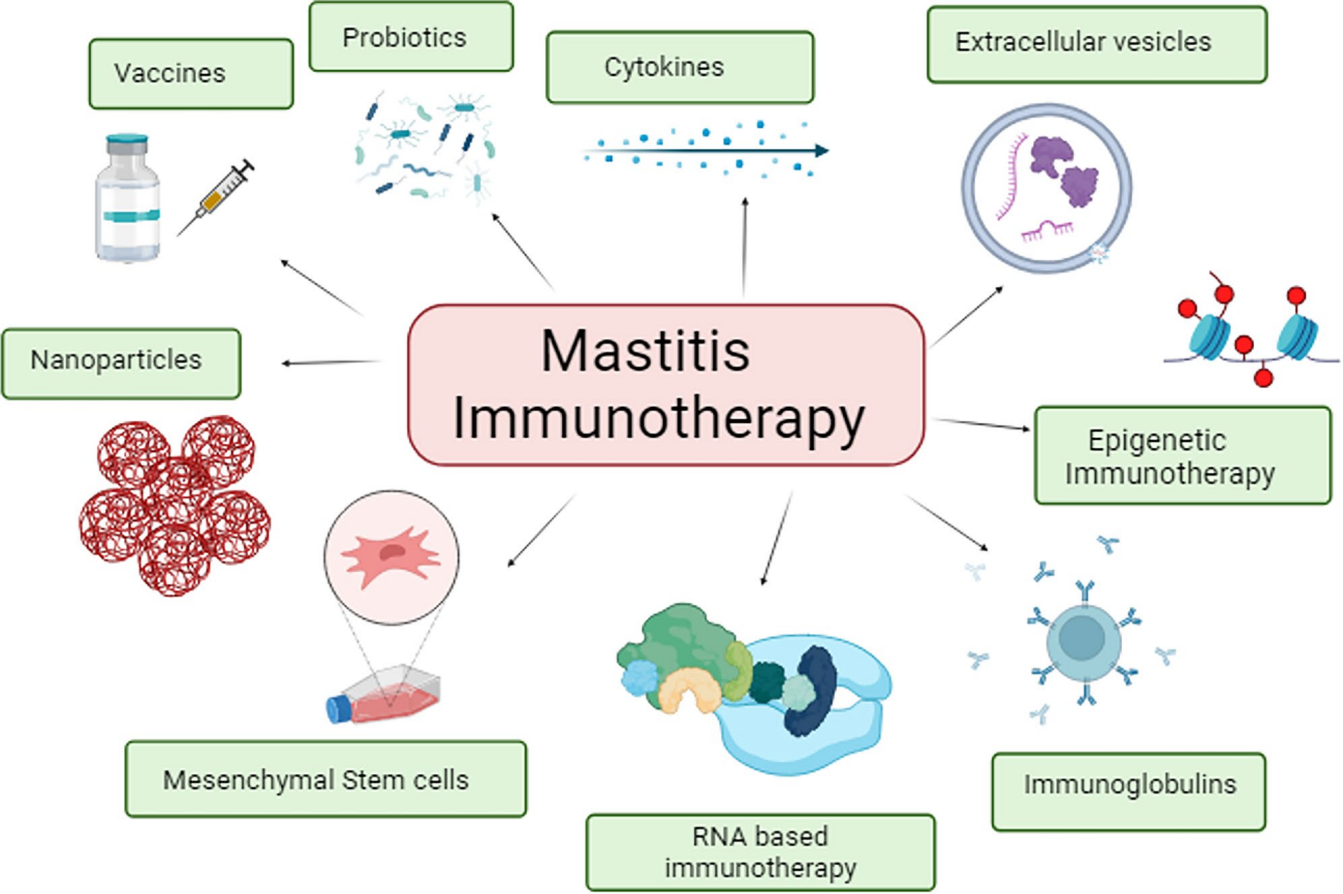
Mastitis immunotherapy strategies.

Immunotherapy, an immunological treatment strategy has been an active area of investigation in the last decade. It consists of manipulation of the immune system to produce specific factors that function to strengthen the natural host defenses against infections (Schwab 2008; Qadri et al. 2023). They can be classified as active or passive immunotherapies based on their mode of action. Active immunotherapy uses virulence aspects to stimulate effectors (T-cells or the humoral response) and to trigger host immunological memory components while passive immunotherapies include constituents produced *ex vivo* and administered to host/patient. Consequently, various immunotherapeutic and other regimens, such as vaccination, T/B-cell immunotherapy, RNA immunotherapy, epigenetic immunotherapy, bacteriophages, bacteriocins, stem cell therapy, antimicrobial peptides, native secretory factors, nanoparticle technology-based therapy, dry cow therapy, and probiotics (Figure 4), have been evaluated for their efficacy and potential application in the management of both clinical and subclinical forms of mastitis. Some of these therapies are presented in more details below.

### 3.1. Vaccination

Efficacious vaccines are of particular interest for the management of mastitis. First generation vaccines which include the classical inactivated (IV) and modified-live vaccines (MLV) have similar advantages in humans and animals. Since inactivated vaccines primarily present antigens through the MHC-II route to trigger humoral immune responses, they are both relatively safe and affordable. Pathogens that require a robust cell-mediated reaction on the other hand, can avoid the pressure created by the classical inactivated vaccines (Castelani et al. 2019; Pinheiro Machado et al. 2019). By triggering both MHC-I and MHC-II pathways, modified-live vaccines successfully multiply within the host and evoke protective immunity. Modified-live vaccines, on the other hand, are not recommended in severely immunocompromised individuals who are at high risk of infection (Arvas 2014). These aforementioned disadvantages were the catalysts for the development of second and third generation vaccines.

Subunit components, synthetic proteins, or conjugated/recombinant antigens are examples of second-generation vaccines. These vaccines are presented on MHC-II complexes because they are recognized by antigen-presenting cells through the intravesicular pathway. Viral-vector platforms, gene-based (DNA and RNA), and live or inactivated chimera vaccines are examples of third generation vaccines. DNA vaccines act as PAMPs and cause humoral and cell-mediated reactions, which reduces the need for adjuvant (Mogensen 2009; Li et al. 2013; Kyriakis 2015). A strong MHC-I mediated CD8^+^T cell response is induced by Plasmid-DNA and RNA vaccines (Sahin and Karikó 2014).

In the past decades, several vaccines have been developed for use in controlling mastitis (Tables 2 and 3). Despite decades of research however, the efficacy of current vaccines is not acceptable (Rainard et al. 2021). Reverse vaccinology and systems vaccinology are two hypotheses that have been put forth to support methods of combating infections that are resistant to vaccine control (Sahin and Karikó 2014; Rainard et al. 2021). The physiology and immunology of the mammary gland, the type and virulence of the bacteria present, and the potential for inducing sterilizing immunity in the mammary gland against commensal bacteria are some of the factors preventing the development of efficacious vaccines for mastitis. As shown in Table 2, *S. aureus*, *S. uberis*, and *E. coli* are the predominant mastitis pathogens and are the main targets for vaccine research (Bradley et al. 2015; Collado et al. 2016; Ashraf and Imran 2020). Most available commercial vaccines are costly and have also failed to demonstrate sufficient protection (Côté-Gravel and Malouin 2019). Furthermore, because numerous microorganisms contribute to the development of mastitis infection, vaccination is ineffective against mastitis. Therefore, more efficacious vaccines for mastitis management are needed.

**Table 2.** Mastitis vaccine trials in cows.

Vaccine	Efficacy	Reference
*E. coli J5 bacterins	Reduced coliform mastitis severity in the field, but minimal effect in experimental infections	Gonzalez et al. (1989); Hogan et al. (1992); Hill (1991)
*E. coli* J5 bacterin with killed S. aureus (StartVac®, Hipra)	Under field studies, reduced severity of mastitis	Bradley et al. (2015)
*E. coli* enterobactin FepA or siderophore receptor FecA	Under *in-vitro* conditions, reduced bacterial growth	Lin et al. (1999)
*S. aureus* bacterins and toxoid or bacterial lysate	Mastitis incidence and severity are reduced	Middleton et al. (2006); Middleton et al. (2009)
*S. aureus* protein A	Enhanced spontaneous cure following experimental challenge	Pankey et al. 1985)
*S. aureus* FnBP and ClfA	Enhanced spontaneous cure following experimental challenge	Shkreta et al. (2004)
*S. uberis* slime preparation (UBAC®, Hipra)	Reduction in the number of clinical mastitis cases and milk production losses	Collado et al. (2018)
*Klebsiella* siderophore receptors and porin proteins (KlebVax™)	Milk production was somewhat increased, and the risk of coliform mastitis was slightly decreased	Gorden et al. (2018); Tomazi et al. (2021)

*E. coli, Escherichia coli; S. aureus, Staphylococcus aureus, S. uberis, Streptococcus uberis; FepA, ferric enterobactin receptor; FecA, ferric citrate receptor; FnBP, fibronectin-binding protein; ClfA, clumping factor A.

**Table 3.** Available commercial mastitis vaccines.

Name of the vaccine	References
*E. coli mutant core antigen J5 (Startvac, Hipra, Spain)	Ismail (2017)
*E. coli* rough mutant O111:B4 bacteria containing vaccine	Wilson et al. (2009)
*S. aureus,* and *S. agalactiae* consists of either the whole organism (cellular lysates, inactive and attenuated vaccines) or subunits (toxins, polysaccharides, and surface proteins)	Ismail (2017)
*S. aureus* strain SP 140	Freick et al. (2016)
*S. aureus* JR3 cells and SM capsule of the strain *S. aureus* 2286	Slobodanka et al. (2008)
Trivalent vaccine contained *S. aureus* capsular polysaccharide type 5 (T5), 8 (T8), and 336 (T336)	Lee et al. (2005)
*Staphylococcal enterotoxin* Type C mutant vaccine (MastaVac)	Chang et al. (2008)
Herd-specific autovaccine (Best Vac)	Freick et al. (2016)
Polyvalent herd-specific autovaccine containing *S. aureus* strain SAU 7 and *S. agalactiae* strain SAG 3	Magaš et al. (2013)

*E. coli, Escherichia coli; S. aureus, Staphylococcus aureus; S. agalactiae, Streptococcus agalactiae.

For optimal vaccine efficacy, efficient delivery systems are required to activate a strong immune response. Nanoparticles (Song et al. 2014) and live bacterial vectors (Yurina 2018) are promising delivery systems for DNA vaccines. Current development strategies for vaccines mainly rely on small animal models, and commercial DNA vaccines against bovine infectious diseases are still in their preliminary phases. Large animal models have limited plasmid delivery and expression which impacts the weak immunogenicity of the antigen. Clinical trials have shown the ability of exosome-based vaccines in recruitment and activation of innate immunity. However, loading extracellular vesicles (EVs) with specific antigens or drugs for a more efficient cargo delivery is vital for exosome-based vaccine investigations (Santos and Almeida 2021). Use of exosomes have been proposed as a cell-free vaccination platform against various infectious diseases (Sotillo et al. 2016; Samoil et al. 2018). Extracellular vesicles derived from Gram-positive bacteria have recently gained attention as a potential vaccine platform for several infectious diseases. For example, *S. aureus* EVs were modified to serve as vaccine candidates (Wang et al. 2018).

### 3.2. T cell immunotherapies

The primary pathways that induce either immunity or tolerance are interactions between T cells and dendritic cells (Steinman 2012). The immune response and the efficiency of any particular outcome are rather an amalgam of numerous cellular interactions. T cells are fundamental to the mechanism of the majority of immunotherapies that have received clinical approval for human health management (Mitra et al. 2023). T cells are powerful effectors of the immune response and are distinguished by dynamic changes in the ratios of CD4:CD8 T cells, T effector (Teff) to regulatory T cells (Treg), and canonical T cell differentiation states, such as naive T, helper T cell subsets, Teff, tissue-resident memory cells (TRM), and exhausted T cells (Tex). Macrophages are known to have a significant role in the bovine mammary gland as cells that process and present antigens to T-cells. While CD4 + T-cells are recruited from the blood and become the predominate phenotype in milk under pathological settings, CD8+ T cells are primarily detected in the milk of healthy glands (Ezzat Alnakip et al. 2014). During bovine *S. aureus* infection, increased CD8^+^ T-lymphocyte levels in the bovine milk was observed (Park et al. 1992). Later, it was discovered that CD8+ T-cells were in charge of inhibiting milk CD4+ T-cells proliferative response (Park et al. 1993). Previous research has revealed that gamma-delta T cells (γδ T) cells contribute in the host inflammatory response (Spinozzi et al. 1998; Zuany-Amorim et al. 1998). gd T cells were also demonstrated to regulate local cellular traffic by promoting the influx of lymphocytes and monocytes and thus, limiting the availability of inflammatory cells which do not act as an anti-infection defense but rather harm tissue (D’Souza et al. 1997). It was also reported that CD81 gd T subset cells in bovine milk down regulated the response of CD41 T cells to staphylococcal antigens (Park et al. 1993, 1994).

### 3.3. B cell immunotherapy

B cells activate the host’s inherent innate immunity to fight off pathogenic infection, allowing it to resolve on its own. The IgG-dependent antibody production pathway produces antibodies upon detection of the antigen (Nimmerjahn 2014) which helps the host to develop resistance against the causative pathogen. Therefore, IgG binding to bacteria allows the clearance of pathogenic organism and its associated toxic products from the body. TIL-B lymphocytes (tumor-infiltrating B lymphocytes) are thought to be better immune system antigen-presenting cells (APCs) (Nelson 2010). Activated B cells can act as APCs for T cells (both CD4+ and CD8+). They have an advantage over dendritic cells (DCs) in that they specifically present the cognate antigen (Ag) acquired *via* surface immunoglobulin (Ig) molecules, which can be used as a therapy or adjuvant therapeutic aid (Leitner et al. 2013). This occurs even at a minimal concentration of antigen (Kurt-Jones et al. 1988). Dendritic cells require initial T cell priming, and B cells encourage T cell growth and memory development (Milne et al. 2009; Tobón et al. 2013; Rodriguez-Pinto and Saravia 2014). Additionally, B cells aid in the development of Th1 cytotoxic T-cells and improve T-cell mediated immunity. In cattle, Y-complex (anti-mastitis bacteria antibodies along with a phagocytosis enhancer) was found as effective as antibiotics, and superior to NSAID (a non-steroidal anti-inflammatory drug), in bacterial elimination (Leitner et al. 2013). Injection of Interleukin-2 (IL-2) showed increase of several milk markers which are related to white blood cell and epithelial cell functions including serum amyloid A (SAA), lactoferrin, SCC and NAGase (Zecconi et al. 2009). Given that B-lymphocytes have a dynamic nature that can be selectively activated or repressed by targeted therapy, this opens up an intriguing option for employing immunotherapy for the treatment and control of mastitis.

### 3.4. RNA-based immunotherapy

The altered expression or dysfunction of many genes has been associated with human and animal diseases. A plethora of investigations have reported altered expression of proinflammatory cytokines, anti-inflammatory cytokines and other classes of immune genes in relation to mastitis caused by various pathogens (Milne et al. 2009; Wang et al. 2013; Song et al. 2016; Han 2019; Chen et al. 2021; Niedziela et al. 2021; Gao et al. 2023). Moreover, knowledge of the roles of genes during disease has been used to design messenger RNA (mRNA) therapeutics with high specificity, validity and safety for the management of many human diseases (Beck et al. 2021; Deng et al. 2022; Zhu et al. 2022). The types of RNA-based immunotherapies developed to control human diseases with varying degrees of success include antisense oligonucleotides, RNA interference, mRNAs and mRNA vaccine, CRISPR-based genome editing and aptamer, amongst others (Zhu et al. 2022; Delaunay et al. 2023).

Messenger RNA immunotherapy has been studied as a potential treatment to reduce inflammation and promote tissue repair in diseased animals. For instance, a study on cows with Johne’s disease (JD) highlighted the functional importance of the interleukin-10 receptor alpha (IL10RA) gene as an immunoregulatory cytokine during the pathogenesis of inflammatory disorder (Mallikarjunappa et al. 2020). According to the findings, CRISPR/cas9 deletion of IL10RA boosted the expression of pro-inflammatory cytokine genes (TNF-α, IL1A, IL1B, and IL6), decreased the expression of SOCS3 (a negative regulator of pro-inflammatory cytokine signaling), and increased the protein expression of inflammatory cytokines (TNF-α and IL-6) and chemokines (IL-8, CCL2 and CCL4) (Mallikarjunappa et al. 2020). The authors concluded that IL10RA elicited an anti-inflammatory response and performed immunoregulatory role in Johne’s diseased cows (Mallikarjunappa et al. 2020). In recent years, third generation mRNA vaccines as well as DNA and recombinant viral vector vaccines, which induce both cellular and humoral immune responses have been developed for livestock species (Aida et al. 2021). Such mRNA vaccines employ an mRNA segment encoding antigens contained in vesicle carriers. After being introduced to the host’s cell, the RNA is translated directly, leading to the production of the desired antigen.

The regulation of mammalian genes has become a much more urgent subject than the fundamental tenet of molecular biology in recent years since less than 2% of the mammalian genome is known to code for proteins (Grinman et al. 2019). Therefore, studies have shown the involvement of various regulatory non-coding RNAs (ncRNAs) in livestock diseases (Do et al. 2021; Oyelami et al. 2022) which points to their potential immunotherapeutic functions. The class of non-coding RNAs known as regulatory ncRNAs includes microRNA (miRNA), PIWI-interacting RNA (piRNA), small nucleolar RNA (snoRNA), long non-coding RNA (lncRNA), and others. The potential and promise of regulatory ncRNA immunotherapy in the treatment of human diseases have been examined (Cortez et al. 2019; Vishnubalaji et al. 2020; Chen et al. 2021; Di Martino et al. 2021; Ma et al. 2022; Xiao et al. 2023). In farm animals, the involvement of miRNAs, lncRNAs and circRNAs in regulating farm animal diseases (e.g. mastitis, foot and mouth disease, Johne’s disease) and their potential as candidates for disease management were recently reviewed (Kosinska-Selbi et al. 2020; Dysin et al. 2021; Do et al. 2021; Oyelami et al. 2022). In the mammary gland, miRNAs have been demonstrated to control the host immune system, promote tissue healing, and reduce inflammation. For instance, Jin and colleagues investigated how mastitis infections (*E. coli* and *S. aureus*) affected the host’s ability to defend itself *via* miRNAs (Jin et al. 2014). Gram-negative *E. coli* strain P4 or Gram-positive *S. aureus* strain Smith CP bacteria were used to infect bMECs (MAC-T cells). The authors found that five differentially expressed (DE) miRNAs (bta-miR-184, miR-148, miR-486, let-7a-5p, and miR-24-3p) were specific to *E. coli*, while four DE miRNAs (miR-23a, bta-miR-2339, miR-499, and miR-99b) were specific to *S. aureus* (Jin et al. 2014). The target genes of the dysregulated miRNAs were also found to be enriched in a number of pathways, including those involving the cellular processes, signal transduction, immune system, and diseases, among others (Jin et al. 2014). In another study, infection of primary bMECs with *S. uberis* (strain 0140 J) revealed the up-regulation of let-7b and miR-98 and the down regulation of miR-15a, miR-26a-2, miR-29a, miR-29b-2, miR-29c, miR-29e, miR-100, miR-17, miR-29b-1, and miR-193a (Lawless et al. 2013). Significantly more innate immunity-related genes, including those involved in the MAPK, JAK-STAT, and other cytokine signaling pathways, were predicted targets of the DE miRNAs (Lawless et al. 2013). Several reports have shown that LncRNAs also regulate the host immune system during mastitis infection (Yang et al. 2017; Wang et al. 2019; Mi et al. 2021). In a recent study, Mi and colleagues associated two lnRNAs (PRANCR and TNK2–AS1) with bovine *S. aureus* mastitis (Mi et al. 2021). According to the study, PRANCR controls the mRNA expression of selectin P ligand (SELPLG) and integrin beta 2 (ITGB2) genes that are involved in *S. aureus* infection pathways and also promotes apoptosis in MAC-T cells, suggesting roles for PRANCR and TNK2-AS1 in immune regulation during bovine *S. aureus* mastitis. Using computational analyses to identify lncRNA target genes related with bovine mastitis immune response, Tucker et al. reported two lncRNAs (ONBTAT027932.1 and XR_003029725.1) which targets several genes that are involved in regulating lipopolysaccharide-mediated signaling pathways, chemokine (C-X-C motif) ligand 2 production, and IL-23 production (Tucker et al. 2021). LncRNAs can also be used to strengthen the host defense mechanism or to encourage bacterial invasion or replication inside host cells. Imamura et al. observed that the silencing of lncRNA NEAT1v2 or enhancer RNA eRNA07573 after Salmonella infection in HeLa cells decreased the survival rates of cells while studying the potential involvement of lncRNA during *Salmonella enterica serovar typhimurium* virulent strain χ3306 infection (Imamura et al. 2018).

The aforementioned reports are indications that the involvement of both mRNA and regulatory ncRNA in modulating the host immune response and disease pathogenesis shows promise for their development as immunotherapies for the management of mastitis.

### 3.5. Cytokine immunotherapy

Recombinant DNA technology advancements have made it possible to produce vast amounts of animal cytokines for use as cytokine immunotherapy to treat mastitis. Based on species cross-reactivity between bovine and human cytokines, studies have been done wherein recombinant cytokines of human origin have been administered to cattle. In an experimental mastitis model, recombinant human granulocyte colony-stimulating factor (rhG-CSF) was administered to lactating dairy cows by subcutaneous injection. When compared to placebo-treated controls, experimental *S. aureus* challenge-induced mastitis was reduced by 47% (Nickerson et al. 1989). Recombinant human granulocyte colony-stimulating factor reportedly recruited neutrophils into the mammary gland prior to the infection. However, no prevention or treatment of *S. aureus* was seen when rhG-CSF was infused through intramammary route. It has been demonstrated that infusing bovine IL-2 through the intra-mammary route improves the humoral and cellular immune response in *S. aureus* infected animals. Interleukin-2 given as a preventative measure shielded the mammary gland from a future *S. aureus* infection (Daley et al. 1991). It was also reported that prolonged intramammary infusion of IL-8 elicited inflammatory responses such as prolonged secretion of elastase, IL-8 and inflammatory lactoferrin derived peptides, implicated in the pathogenesis of *S. aureus* dry-period mastitis (Watanabe et al. 2012). However, therapeutic administration of cytokines was less efficient at eliminating preexisting infection since activation in the mastitic gland is mainly achieved through bacterial components and secondary host signals. Exogeneous cytokine administration merely mimics normal host reactions to infectious agents, meanwhile the phagocytic cell function is significantly depressed in normal milk PMN when compared with peripheral blood PMN (Daley et al. 1991). Thus, homologous cytokines use in combination with other strategies may improve the overall efficiency of these therapeutic agents (Daley et al. 1992). Interferon-gamma (IFN-**γ**) was also found to reduce endotoxemia-related mortality and morbidity brought on by bacterial toxins. When compared to placebo, cows given intramammarily IFN-**γ** prior to an *E. coli* challenge resulted in fewer infected quarters, minor clinical scores, and illnesses that lasted for a shorter period of time (Sordillo and Babiuk 1991). More cows in the placebo group displayed clinical mastitis (70%) when compared with cows treated with lFN-**γ** (16.7%) (Sordillo and Babiuk 1991).

Recombinant cytokines can change the course of mastitis infection in individuals with weakened immune systems. They are able to accomplish this by a combination of the recruitment of effector cells to the mammary gland, improved phagocytic cell clearance of pathogens, and control of acute inflammatory responses.

### 3.6. Immunoglobulins

Egg yolk immunoglobulins (IgY) have been used in several *in vitro* studies for treating mastitis (Zhen et al. 2008; Wang et al. 2011). Egg yolk immunoglobulin is produced by immunizing hens with formaldehyde killed bacteria (*E. coli and S. aureus*) in a long-standing immunization response. The increased phagocytic activity of egg yolk immunoglobulin against bacteria suggests that it could be used as a mastitis therapeutic agent (Zhen et al. 2008). According to a recent report, *S. uberis* opsonized with affinity purified anti-recombinant *S. uberis* adhesion molecule (anti-rSUAM) antibodies or hyperimmune sera in treated animals outperformed untreated animals (Almeida et al. 2015). This result showed improved protection against *S. uberis* by antibodies which prevented adherence and inhibition of pathogen entry into the gland. Lower milk bacterial counts as well as mild to undetectable symptoms of mastitis were detected in the antibodies treated animals.

### 3.7. Epigenetic immunotherapy

Epigenetic processes are a multi-layered regulatory system that alters the gene expression patterns of cells in response to stimuli (such as pathogens, diet, environmental contaminants, etc.) without changing the core DNA sequence. The epigenetic mechanisms involve chemically modifying nucleic acids (DNA, RNA), and histones, as well as chromatin accessibility, among others. A plethora of human and animal studies have shown diverse functions for epigenetic mechanisms in a variety of biological processes, including growth, development, metabolism, and health (Ibeagha-Awemu and Zhao 2015; Zoghbi and Beaudet 2016; Ibeagha-Awemu and Khatib 2023). In particular, modifications to the epigenome have been associated with health and disease in livestock and humans, and in the dynamic control of immunological reactions to infections and other stressors (Emam et al. 2019; Safi-Stibler and Gabory 2020; Wang and Ibeagha-Awemu 2020). As an illustration, studies on DNA methylation and the immune response in cattle have reported the methylation of immune-related genes and overall alteration of DNA methylation patterns in response to numerous pathogens causing livestock diseases (Wang and Ibeagha-Awemu 2020), pointing to its potential use in immunotherapy.

Epigenetic immunotherapy which use epigenetic alteration information of immune cells to manipulate host immune system to fight infections has been successfully applied in human health management (e.g. cancer management), either alone or in combination with other therapies or to make more efficacious epigenetic drugs (Topper et al. 2020; Tien et al. 2023; Yang et al. 2023). In particular, the combination of immune regimens and epigenetic therapy, such as combining immune checkpoint blockade with epigenetic agents is a winning strategy used to addressed the shortcomings of immunotherapy in cancer management (Gomez et al. 2020; Liu et al. 2022).

### 3.8. Stem cell therapy

The problem of mammary tissue regeneration, whose integrity is vital of milk production, has not been addressed till now. Regenerative medicine puts to use stem cells or their secretome to treat disease, and thus represents a potential tool in the treatment of bovine mastitis and deserves attention.

Mesenchymal stem cells (MSCs) are situated in several tissues of the body and are multipotent progenitor cells. They possess the ability to differentiate into mesodermal lineages such as osteogenic, adipogenic, and chondrogenic lineages (Caplan 2017). According to a recent study, inflammatory interferon γ (IFNγ) activation of bovine foetal adipose tissue-derived mesenchymal stem cells (AT-MSCs) led to the up-regulation of immunomodulatory factors such as indolamine 2,3-dioxygenase (IDO) and IL-6 ^144^. Additionally, *in vitro* conditions allowed bovine fetal AT-MSCs to stimulate angiogenesis through the production of vascular endothelial growth factor (VEGF) and angiopoietin 1 (ANGPT1) (Jervis et al. 2019). Bovine foetal AT-MSC-conditioned medium has an anti-proliferative impact on *S. aureus. In vitro* antibacterial effect of fetal bovine MSC is mediated through antibacterial peptides defensin 1 (DEF1) and NK Lysin (Cahuascanco et al. 2019).

Due to MSCs decreased immunogenicity which enables immune evasion and MSC allogenic transplantation, they may also be used in cell therapy (Ankrum et al. 2014). T-cell costimulatory markers CD80 absence and decreased expression of MHC-I and II) suggests that bovine fetal AT-MSCs are immunological evasive (Huaman et al. 2019). These enable the use of the bovine fetal AT-MScs as an allogenic therapeutic approach by preventing host immune system recognition in recipients.

Mesenchymal stem cells have antibacterial action because they produce substances that impede bacterial development (Cahuascanco et al. 2019). Bone marrow derived MSCs have shown *in vivo* antibacterial activity against methicillin-resistant *S. aureus* (MRSA) in a rat model (Chow et al. 2020). Either the innate immune response of MSC is enhanced or certain anti-microbial peptides expression is enhanced to ensure rapid bacterial clearance (Yuan et al. 2014). This property has been utilized in bovine mastitis therapy. When compared to untreated cows, intra-mammary inoculation of allogeneic AT-MSCs allegedly lowered the bacterial count in clinical mastitic cows (Peralta et al. 2020). Furthermore, in healthy cows, intramammary injection was not related with clinical or immunological response (Peralta et al. 2020). Because stem cells possess the capability to develop into epithelial, cuboidal/columnar, or myoepithelial cells of the udder tissue, they have been employed to either repair or replace mammary tissues and thus, regenerate the damaged tissue. The risk of rejection and associated negative effects can be reduced by using bovine mammary stem cells (Sharma and Jeong 2013). Capuco et al. (2012) reported that mammary stem cells can be exploited for tissue repair and improvement in milk yield. Understanding cell development in mammary tissue requires the isolation and identification of mammary stem cells (Sharma and Jeong 2013).

### 3.9. Extracellular vesicles and exosome immunotherapy

Exosome is among several molecules referred to as extracellular vesicles (EVs). Extracellular vesicles are membrane vesicles of endosomal (exosomes) and plasma membrane (microvesicles) origin released into the extracellular environment by cells. Extracellular vesicles are vital for intercellular communication because they transport metabolites, proteins, lipids, and nucleic acids between cells. They serve as mediators of local and distant intracellular communication in disease and health and modulate various biological processes. Thus, EVs are considered important vehicles of inter- and intra-species cellular communication as they transfer myriad of biomolecules including genetic information (Celluzzi and Masotti 2016). Bacterial vesicles protein content incorporates factors for virulence, biofilm formation, antibiotic resistance, stress response, bacterial survival, lateral gene transfer, pathogenicity, and inter- and intra-species cooperation and communication (Haurat et al. 2015).

Extracellular vesicles are a new class of nanocarriers that have made it possible to create medical formulations based on EVs for the treatment of a variety of illnesses, including infectious diseases, regenerative disorders, cancer, neurological disorders and autoimmune disorders (Akbari et al. 2020; Tran et al. 2020; Chung et al. 2020; Kumar et al. 2020). Acting as short- and long-distance intracellular communicators, EVs are highly effective drug nanocarriers. Exosomes (30–120 nm), microvesicles (MVs) (50 nm-1m), and apoptotic bodies (500–1000 nm) are all examples of extracellular vesicles (Batrakova and Kim 2015; Vader et al. 2016; Villa et al. 2019). Extracellular vesicles’ cellular membranes contain a variety of cellular adhesion proteins, which facilitate quick cell entrance and effective distribution of therapeutic cargo delivery (Théry et al. 2006; Théry et al. 2009).

*S. aureus* extracellular vesicles have been reported to contain a variety of bacterial proteins, including cell surface proteins and toxins. They can be employed as a novel approach in vaccine development against *S. aureus* since they can effectively elicit an adaptive immune response without the need for adjuvants (Toyofuku et al. 2019; Asano et al. 2021). A study reported inflammatory response of pbMECs to *S. aureus* extracellular vesicles (Saenz-de-Juano et al. 2022). The virulence factors produced by live pathogens to damage cells without being recognized as a pathogenic agent are contained in extracellular vesicles. When they build up sufficiently in the alveolar fluid, they can drastically change the MEC’s immunological reactivity (Saenz-de-Juano et al. 2022).

Exosomes are the EVs which are secreted by cells reflecting the state of the cells and can be isolated from bodily fluids, such as milk, blood, tears, urine, lymph, mucus, semen, saliva, sweat, bronchial lavage, ascitic fluid, and cell supernatant, and they contain stable cell source-specific nucleic acids, mRNAs, noncoding RNAs (ncRNAs), membrane proteins, nuclear proteins, and various metabolites. Exosomes have been used for disease diagnosis, treatment as well as for performing disease mechanism analysis (Ji et al. 2022).

#### 3.9.1. Milk derived exosomes

Exosomes produced during bacterial-induced mastitis by MECs have been studied. Milk contains exosomes but their content and biological functions change during mastitis. The miRNA and protein profiles of MAC-T cell derived exosomes were found to be consistent with exosomes from milk and thus, for studies using milk exosomes, MAC-T cells can be employed (Ogunnaike et al. 2021). Bta-miR-142-5p and bta-miR-223 have been identified by Sun and Cai as potential biomarkers for the early mastitis diagnosis after studying the bovine milk exosomes miRNA expression profile during *S. aureus* infection (Sun et al. 2015; Cai et al. 2018). In another study, 37 miRNAs were found to be differentially expressed in exosomes from milk of cows infected with *S. aureus* vs healthy cows (Ma et al. 2019). Specifically, bta-miR-378 and bta-miR-185 were found to have considerably enhanced expression in exosomes generated from milk of cows infected with *S. aureus*. These miRNAs are known to have associations with multiple health parameters including a role in diseases like hepatic inflammation and fibrosis. Milk exosomes from subclinical mastitic cows were analyzed for three consecutive days for their size and concentration as well as their miRNA cargo. It was found that they persisted for three days and that specific miRNAs in milk exosomes were related with particular physiological states (Saenz-de-Juano et al. 2022). In particular, bta-miR-223-3p had a high expression level and demonstrated great potential for use as a diagnostic marker for subclinical mastitis (Saenz-de-Juano et al. 2022). Exosome cargo sorting in donor cells and the development of new exosome-mediated cell contact models are required for the effective implementation of exosome therapy.

#### 3.9.2. Exosomes as vehicles for drug delivery in mastitis treatment

Exosomes derived from milk have been shown to be absorbed by an array of cells (Izumi et al. 2015; Ogunnaike et al. 2021) and can be used as delivery vehicles for drugs, short interfering RNA (siRNA) and miRNA (Del Pozo-Acebo et al. 2021; Luo et al. 2021). The anti-inflammatory, antiviral and anticancer activities (Aarts et al. 2021; Yenuganti et al. 2022; Babaker et al. 2022) of milk-derived exosomes have been characterized. Exosomes as drug delivery vehicles reportedly serves many advantages as they are safe without any induced immunogenicity or cytotoxicity (Somiya et al. 2018; Ross et al. 2021), have ability to target effectively (Zempleni et al. 2019), have capability to pass the body barrier systems such as blood-brain barrier (Manca et al. 2018), have ability to increase the oral availability of drugs (Betker et al. 2019), are stable against extreme stimuli (Izumi et al. 2012; Ngu et al. 2022), have standardized preparation process and are cost-effective (Sedykh et al. 2020; Li et al. 2022). In a recent study, it was discovered that dairy cows with subclinical mastitis responded favorably to exosomes produced from mesenchymal cell-derived allogeneic umbilical cord blood (Ghai et al. 2022).

### 3.10. Nanoparticle-based therapy

Nanoparticles improve the antibacterial activity by increasing the active absorption of substances by phagocytes. They have been utilized to combat a number of microorganisms with multi-drug resistance (Yu et al. 2018; Castelani et al. 2019). Nanoparticles have been assessed for mastitis treatment with significant results (Kalińska et al. 2019; Orellano et al. 2019). The difficulty of treating *S. aureus* infections with traditional therapy is because of its unusual facultative intracellular parasitism, potent pathogenicity, development of biofilms, and rising potential for antimicrobial resistance development. For *S. aureus* mastitis treatment, nanoparticle-based therapy approaches are becoming more and more common. These include nanogels, liposomes, inorganic nanoparticles, polymeric nanoparticles, and solid lipid nanoparticles (Algharib et al. 2020). Silver and copper nanoparticles (Kalińska et al. 2019), propolis nanoparticles (Pinheiro Machado et al. 2019), chitosan nanoparticles (Orellano et al. 2019), and cationic nisin-lipid nanoparticles (Castelani et al. 2019) have shown positive results in mastitis management. Tilmicosin nanoparticles have been reported to exhibit prolonged anti-bacterial activity against *S. agalactiae* and *S. aureus* (Zhu et al. 2018). Similarly, amoxicillin nanoparticles enhance the post-antibiotic effect as well as reduce the dosing interval when used to treat bovine mastitis (Yang et al. 2009). Silver and gold nanoparticles have also found use against *S. aureus* mastitis (Elbehiry et al. 2019). Metal nanoparticles like copper and silver were reported to reduce the *in vitro* survival of *S. aureus* and *E. coli* without causing harmful effects on mammary cells (Kalińska et al. 2019). Chitosan nanoparticles (Ch-NPs) have also been used as therapeutics for bovine mastitis (Orellano et al. 2019). The expression of NF-κB-p65 (nuclear factor kappa-light-chain-enhancer of activated B cells), UCHL-1 (ubiquitin carboxyl-terminal hydrolase-1), and SREBP-1c (sterol response element-binding protein-1c), as well as SCC and total microbial count, were all significantly reduced by α-linolenic acid-based intramammary nanosuspension (ALA-NS) (Yadav et al. 2020).

Plant based nanoparticles have also been used for mastitis management. Silver nanoparticles and quercetin from plants displayed potent antibacterial and anti-biofilm activity against multi-drug resistant *E. coli* strains (Yu et al. 2018). Curcumin is a polyphenol derived from turmeric that has anti-inflammatory properties. However, due to its rapid elimination from the body, its bioavailability is poor. Suresh et al. (Suresh et al. 2018) formulated curcumin nanoparticles which improved its oral bioavailability while lowering pro-inflammatory mediators in *S. aureus* infected mammary tissue in a mouse model. Aloin is a major constituent of Aloe vera which has been used to synthesize nano-silver particles. In an experimental murine mastitis model, aloin was discovered to have strong anti-bacterial action against *S. aureus (*Kumar et al. 2013). Using nanoparticles approach is a safe, effective and contamination free mode to overcome multi-drug resistant bacteria in mastitis management.

### 3.11. Microbial administration

The microbiota of cattle plays essential roles in the extraction of energy and nutrients from food and also in the development of the immune system and health maintenance (Uyeno et al. 2015; Celi et al. 2017; Malmuthuge and Guan 2017) While it has become increasingly clear that microbial populations play key roles in health and disease, the disruption of this ecosystem can trigger health disorders including mastitis. Moreover, polymicrobial and correlative studies have reported alterations in milk microbial community suggesting the existence of a causal relationship between the milk microbiota and host phenotype in mastitis (Boix-Amorós et al. 2020; Couvillion et al. 2023). Recently, it was reported that fecal microbiota transplantation led to dysfunctional microbiota in the gut as well as caused mastitis in animal models (Ma et al. 2018; Hoque et al. 2022). Meanwhile administration of probiotics and their metabolites such as short chain fatty acids (Hu et al. 2019) have potential in treating and preventing mastitis.

Lactic acid bacteria and several other microorganisms have been evaluated for their probiotic activity (Dhama et al. 2016). Lactic acid bacteria reportedly have potent immunomodulatory activity by stimulating local and systemic immune response when used as feed supplements, teat dip, and intramammary inoculation (Pellegrino et al. 2017; Yu et al. 2017). Lactic acid bacteria incorporation in animal feed has been found effective in preventing mastitis in cattle (Pellegrino et al. 2017). During the dry period, intramammary inoculation of *L. lactis* subsp. lactis CRL 1655 and *L. perolens* CRL 1724 led to an increase in blood and milk immunoglobulin (IgG isotypes). Lactic acid bacteria (e.g. *Lactobacillus brevis* 1595, *L. brevis* 1597 and *L. plantarum)* colonize the udder, form a protective biofilm and inhibits the growth of mastitis-causing pathogens (Rainard et al. 2018; Wallis et al. 2018). A novel probiotic lactobacilli-based teat dip disinfectant was found to be superior in reducing the SCC of cows (Yu et al. 2018). *L. casei* BL23 was reported to modulate the innate immune response in *S. aureus* stimulated bovine mammary epithelial cells (Souza et al. 2018). In a recent study, dietary supplementation with the commensal bacteria Roseburia was found to alleviate gut-dysbiosis-induced mastitis in a mice model (Zhao et al. 2022). *Saccharomyces cerevisae* extract infusion into mammary gland during the dry period resulted in an increased activity of immune cells in the gland. Anti-recombinant *S. uberis* adhesion molecule (SUAM) antibodies administration showed mild to undetectable symptoms of mastitis in dairy cows (Almeida et al. 2015).

While the mechanisms by which probiotics contributes to the treatment of mastitis are not yet clear, they have been found to alter the teat apex microbiota preventing the colonization of the teat canal by pathogens (Rainard et al. 2018).

## 4. Research gaps and future perspectives

Mastitis immunotherapy holds prodigious potential for the treatment and prevention of mastitis, in addition to having the advantage of serving as an alternative to antibiotic use in dairy production. The need for alternatives to antibiotics use in livestock production is presently a world health priority, as more and more resistant microbes are emerging with potential to pose serious health problems for livestock, humans and the environment. Immunotherapy therefore has the potential to contribute to solving the issue of antimicrobial resistance in the One-Health-Context. While substantial progress has been made in developing mastitis immunotherapies, enhancing their efficacy for the effective management of mastitis is still far-flung, with a myriad of research gaps that must be filled.

Firstly, deep insights into the mechanisms of the host pathogen interaction and the immune response to the myriad of mastitis pathogens must be uncovered and precise immunotherapeutic biomarkers identified and developed. Recent technological developments in cutting-edge sequencing technologies have provided valuable insights into the molecular mechanisms of the host pathogen interaction and furthered understanding of the regulatory mechanisms underlying bovine mastitis (He et al. 2016; Wang and Ibeagha-Awemu 2020; Wang et al. 2020). These studies mostly interrogated single biological layers of the host immune response (e.g. genome or transcriptome, etc.) using single omics tools. Meanwhile, the complex-trait genetic architecture of mastitis is defined by biological processes interacting at multiple layers (e.g. genome, epigenome, transcriptome, proteome, metabolome, microbiome etc.) in response to a myriad of factors. Recently, the integration of two or more biological layers of information or omics (epigenomics, mRNA and ncRNA transcriptomics) resulted in the identification of discriminant biomarkers explaining high variation between cows with *S. aureus* or *S. chromogenes* sub-clinical mastitis compared to healthy controls (Wang et al. 2022; Wang et al. 2023; Laterrière et al. 2023). Thus, it is imperative that multi-omics approaches, which will inform on the complex interactions between the pathogen and host factors, be utilized to provide a more holistic view of the factors in mastitis pathogenesis and identification of precision immunotherapeutic biomarkers.

Deeper understanding of the roles of genes and regulatory molecules (e.g. noncoding RNA (ncRNA) in the host response to pathogens are necessary for informed decisions on the design of effective immunotherapies for the management of mastitis and other livestock diseases. Research developments are required to understand the complex interactions between various mastitis bacterial pathogenesis and how cytokines can modulate their responses. Moreover, mammary gland production of antibacterial proteins for enhancing the resistance to mastitis has been proposed as a prime agricultural application of gene editing technology (Wall et al. 2005). The future of therapeutic applications of cytokines will depend on knowledge on the ability of cytokines to effectively manipulate and regulate mammary immune functions. Epigenetic immunotherapy application in the management of mastitis and other livestock diseases also depends upon knowledge of the altered epigenetics marks and their roles in health and disease, which is currently limited and therefore deserves more research attention.

Artificial intelligence (AI) and machine learning algorithms have recently gained importance in medicine and agriculture due to their ability to recognize patterns within multiple layers of data and unearth correlations that can identify/predict biomarker targets for further development for various purposes (Chen et al. 2023; Guo et al. 2023). In veterinary medicine, and in particular mastitis disease, AI algorithms can unearth correlations in multiple sources of data, such as SCC, milk composition, environmental variables and historical data, etc., to detect early signs of mastitis, implement preventive measures and choose suitable immunotherapies. In particular, AI integration of multi-omics data can identify biomarker targets for development of effective immunotherapies and animal management. Moreover, AI-driven decision support system can also help in making informed decisions regarding the choice of immunotherapies, dosage, and treatment regimens. Therefore, multi-omics approaches and AI integration should form the focus of future research to support the identification of precision biomarkers for mastitis management.

Next generation vaccines, such as DNA and RNA vaccines, could become the next preferred combination partner for long-term mastitis treatment, serving as a platform which is easily combinable with existing immunotherapies. This will open-up innovative treatment options such as combination therapy through multi-target vaccines or vaccination combined with other immunotherapies. However, progress in the discovery of more efficacious vaccines and adjuvants has been slow requiring renewed interest. More attention must also be given to optimize the methods of delivery and to develop safe and effective adjuvants for enhancing the immunotherapy outcome.

Owing to the association of the gut and milk microbiome with mastitis outcome, the role of the microbiota in mitigating mastitis should be given due attention. Exploration of and identification of commensal microbes aiding the mammary immune response to infections will support the development of rational microbiota-based therapeutics. Strategies employed could be by engineering microbiomes, manipulating the gut microbiota, designing probiotics and specific microbial metabolites preparations which will function to augment the efficacy and advance the utilization of precision microbiota therapeutics.

Nanotechnology-based approaches, such as nanoparticle vaccines can enhance vaccine efficacy as well as enable targeted delivery of immunotherapeutic agents to the site of infection, increasing their effectiveness as well as reducing side effects. Nanoparticles and biomaterials can be applied along with immunotherapy, bioengineering and drug delivery to enable programming of the location, pharmacokinetics and co-delivery of immunomodulatory compounds as a targeted therapy for disease. The unique physicochemical properties of nanomaterials have the promise of overcoming antimicrobial resistance by utilizing properties of nanoparticles as delivery vehicles. Nanoparticles also have broad-spectrum antimicrobial potential and will greatly benefit mastitis management. However, knowledge of the application of nanotechnology in mastitis management is currently limited.

It is imperative to evaluate the efficacy of available immunotherapeutic strategies in larger sample sizes and under different conditions. This will include determining the efficacy of various immunomodulators, like vaccines, immunoglobulins, and immunological stimulants in lowering the duration, severity, and incidence of mastitis. Tailored immunotherapeutic interventions will be based on the specific pathogens involved, individual cow’s immune profile, and other factors which will influence mastitis outcome. Development of effective combination therapies, after careful evaluation and optimization of the dose, timing and interactions between the components, will provide synergistic effects and improved outcomes for the cure and prevention of mastitis. To maximize efficacy while minimizing unwanted effects, research is needed to determine the best dose, frequency, and route of administration for various immunotherapeutic approaches. While, some immunotherapeutic approaches have shown promise in laboratory animals or in small-scale trials, there is need for large-scale field trials to evaluate their efficacy under farm conditions.

The potential application of immunotherapeutic strategies in dairy cattle has not been yet realized. To address the menace of AMR, immunotherapy may become increasingly important in the dairy industry in the coming years. Investment in immunotherapy research and development will be vital for the development of effective immunotherapies. However, the affordability of immunotherapy on dairy farms should be considered, including production costs, and the scale of application. Traditional vaccines are a form of immunotherapy and are generally more affordable when compared to some cutting-edge immunotherapies, and are widely used in veterinary medicine, including on dairy farms. However, advanced immunotherapies can be more expensive due to complex manufacturing processes and costs. The initial costs which are associated with testing and developing new immunotherapies may be high but large-scale production and application may decrease cost. Moreover, the added benefit of being an alternative to antimicrobials may encourage wide scale application. Technological advances may also play a role in production costs reduction through improved efficiency of producing and administering immunotherapies. The overall economic impact of mastitis on dairy farms, and impact on human and environment health may influence the overall perceived value and affordability of immunotherapies. Government subsidies, regulatory approvals, and support programs are other measures that may influence affordability and accessibility of immunotherapies.

## 5. Conclusion

The traditional method to treat bovine mastitis is the administration of antimicrobials. However, possible development of antimicrobial resistance in both human and animal pathogens, in addition to the risk of antimicrobial residues in milk calls for development of alternative therapies to treat this global burden. Recent treatment alternatives in the form of various immunotherapies discussed in sections above have shown encouraging results, but there is need for more extensive research to address gaps listed above for informed decisions towards developing more effective and safe immunotherapies for mastitis management.
